# Supplementary material for: An In-House ELISA for Anti-Porcine Circovirus Type 2d (PCV2d) IgG: Analytical Validation and Serological Correlation
Source: Vaccines (Basel). 2025 Jun 19;13(6):657. doi: 10.3390/vaccines13060657 (PMC12197442; doi:10.3390/vaccines13060657)
Supplement: Supplementary file 1 [file vaccines-13-00657-s001.zip › vaccines-3670852-supplementary.pdf]

vaccines

MDPI

Supplementary Materials:

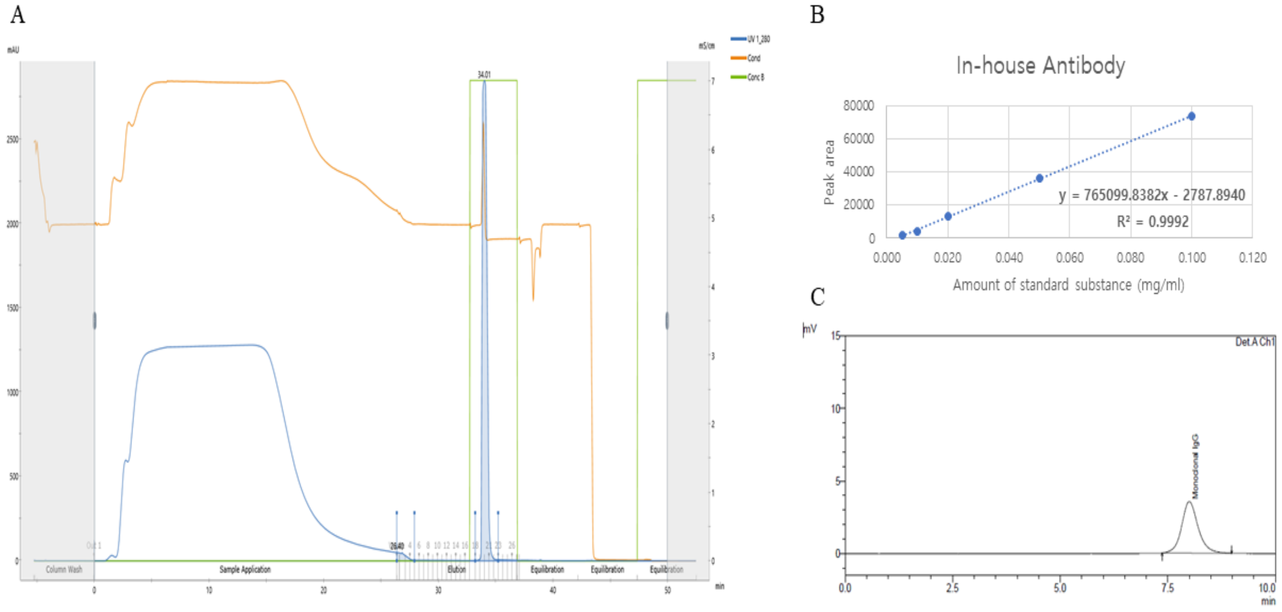

Supplementary Figure S1. Purification, quantification, and quality assessment of in-house polyclonal IgG. (A) Polyclonal IgG was purified from immunized guinea pig antisera using a HiTrap™ Protein A HP column (Cytiva, USA) on the ÄKTA pure™ system. The elution peak was observed between 18 and 21 column volumes, corresponding to the antibody fraction. (B) The in-house IgG was quantified using a standard curve generated from known concentrations, yielding a high linearity ($R^2 = 0.9992$). (C) Size-exclusion chromatography (SEC-HPLC) revealed a single dominant peak at 7.996 min, indicating high monomeric purity of the purified IgG without detectable aggregation or degradation products. No significant interference from elution or equilibration buffers was observed under native conditions.

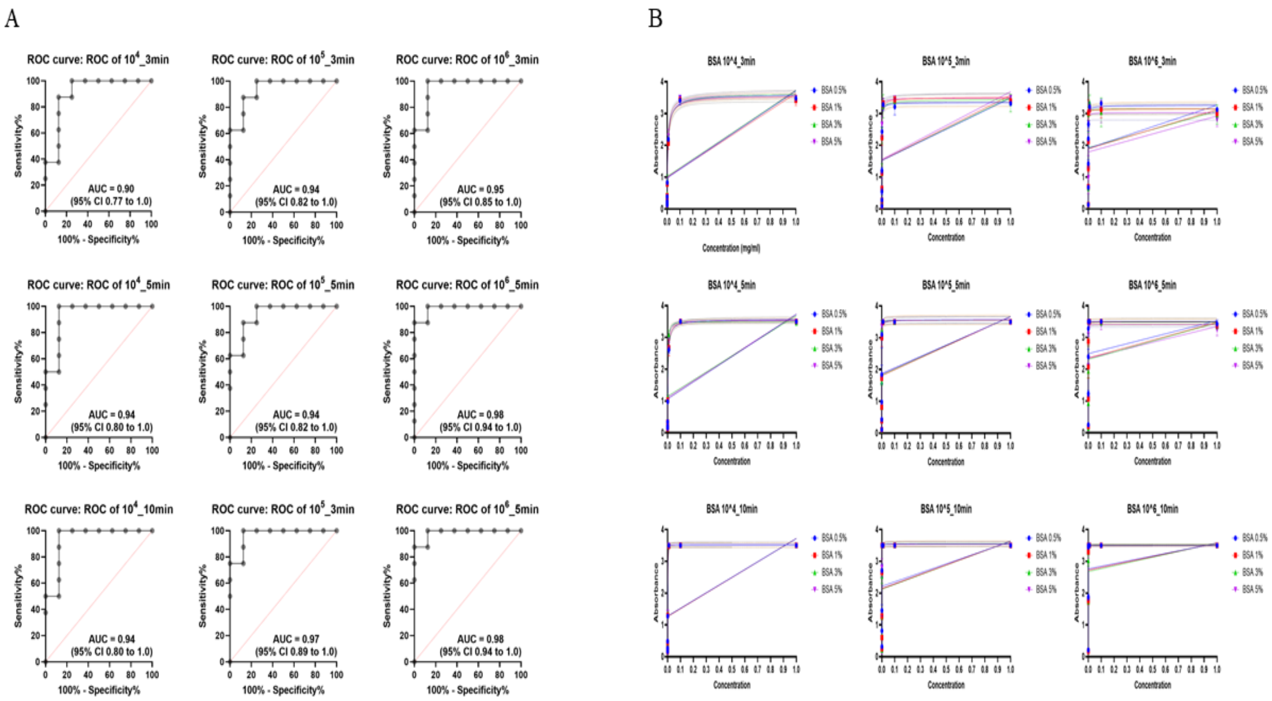

Supplementary Figure S2. ROC curve comparison across BSA conditions. (A) ROC curves generated under varying coating antigen concentrations (10^4, 10^5, 10^6 genomic copies/mL), different percentages of BSA, and different substrate reaction times (3, 5, 10 min). The best condition (10^5 genomic copies//mL, 5% BSA blocking agent, 5 min reaction time) provided an AUC of 0.97. (B) Linear regression analysis for ELISA signal response across BSA concentrations (0.5%, 1%, 3%, 5%) under standardized coating (10^5 genomic copies/mL) and 5 min substrate incubation conditions. BSA at 1% and 3% demonstrated optimal linearity and minimal signal distortion, indicating their suitability for robust assay development.

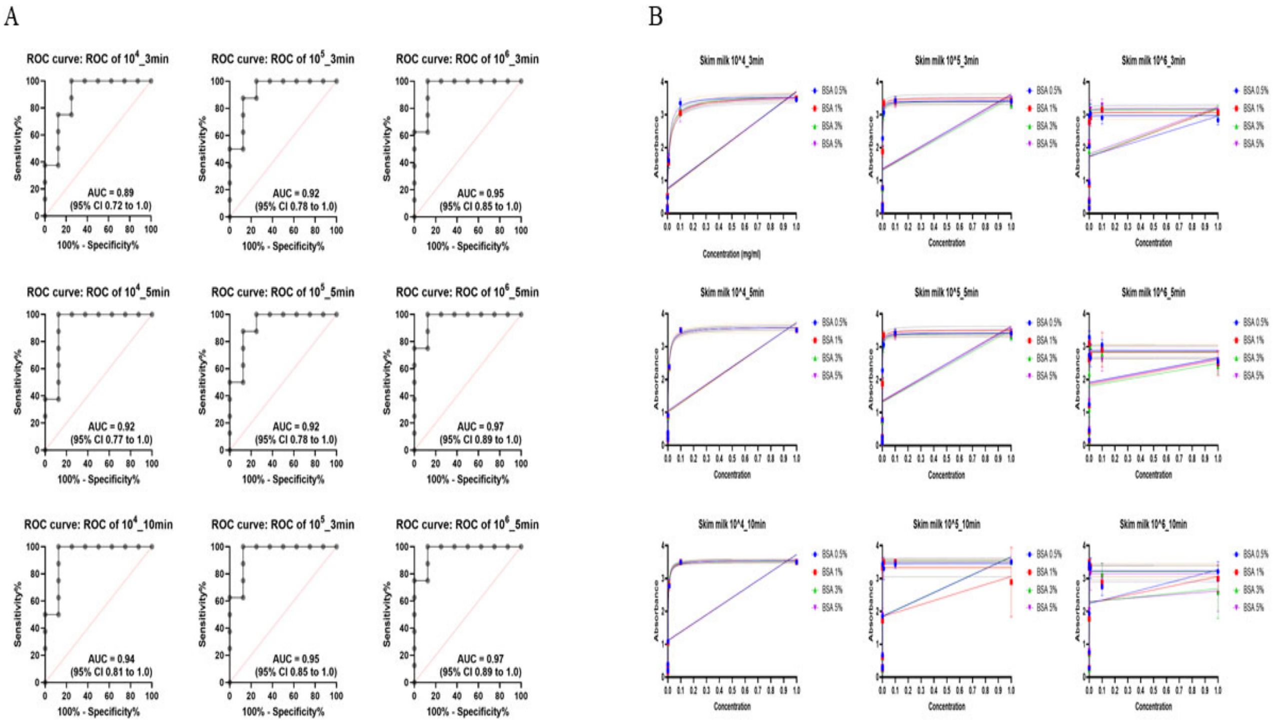

Supplementary Figure S3. ROC curve comparison across four skim milk conditions. (A) ELISA assays were conducted using different percentages of skim milk as the blocking agent under three antigen coating concentrations (10^4, 10^5, and 10^6 genomic copies/mL) and three substrate incubation times (3, 5, and 10 minutes). (B) Absorbance values were measured using an in-house antibody (WG-PCV2d pAb, rabbit anti-PCV2d IgG) across various antigen concentrations. While skim milk generated detectable signals and moderate sensitivity, increased background variability and reduced dynamic range were observed, particularly at lower antigen concentrations. These findings indicate that 5% skim milk is suboptimal compared to BSA for consistent assay performance.
